# The RAGE Inhibitor TTP488 (Azeliragon) Improves Diabetic Bladder Dysfunction in Leptin-Deficient Obese Mice

**DOI:** 10.3390/antiox14070793

**Published:** 2025-06-27

**Authors:** Akila Lara Oliveira, Matheus Leite Medeiros, Antonio Thiago Pereira Campos, Carlos Lenz Cesar, Fabiola Zakia Mónica, Edson Antunes

**Affiliations:** 1 Department of Pharmacology, Faculty of Medical Sciences, University of Campinas (UNICAMP), Campinas 13084-971, SP, Brazil; 2 Department of Physics of Federal Ceara, University of Ceara (UFC), Fortaleza 60440-900, CE, Brazil

**Keywords:** methylglyoxal, void spot assay, methylglyoxal-derived hydroimidazolone, glyoxalase-1, superoxide dismutase, glutathione, collagen

## Abstract

The advanced glycation end product (AGE)–RAGE axis has been implicated in the pathophysiology of diabetic bladder dysfunction (DBD). However, no previous studies have explored the effects of RAGE blockade on this condition. Here, we explored the effects of the selective RAGE inhibitor TTP488 (azeliragon) at the functional and molecular levels of bladder dysfunction in *ob*/*ob* leptin-deficient mice. Female B6.V-Lep ob/JUnib (*ob*/*ob*) and wild-type (WT) C57BL/6 mice were used as lean controls. Treatment with TTP488 in *ob*/*ob* mice resulted in no changes in body weight, fasting glucose, or insulin resistance; however, it reduced total AGE and MG-H1 levels without altering RAGE levels in bladder tissues. TTP488 normalized glyoxalase-1, glutathione reductase, glutathione peroxidase, and superoxide dismutase activities in bladder tissues. Marked increases in collagen intensity were also observed in *ob*/*ob* mice, an effect fully reversed by TTP488 treatment. TTP488 reduced total void volume, volume per void, and ex vivo bladder contractility in response to electrical-field stimulation and carbachol. Our finding that TTP488 mitigates DBD in *ob*/*ob* mice supports the proposal that RAGE blockade could serve as a promising therapeutic strategy for managing DBD.

## 1. Introduction

Diabetes Mellitus (DM) represents a major public health challenge, generating high costs for health systems worldwide. It is estimated that approximately 783 million people will be affected by the disease by 2025 [1]. DM has different phenotypes, with type 2 (T2DM) being the most prevalent, responsible for approximately 90% of cases [2], particularly in middle- and low-income countries, in which the treatment coverage is low [3]. T2DM is associated with overweight and obesity and insulin resistance, as well as with population aging, sedentary lifestyle, inadequate diet, and accelerated urbanization [2]. Diabetic patients often face several cardiometabolic complications, especially in cases of poor glycemic control [3], such as hypertension, dyslipidemia, and kidney diseases [4,5]. Diabetic bladder dysfunction (DBD), also referred to as diabetic cystopathy, is also a condition that affects more than 50% of T2DM-diagnosed individuals, mostly as a result of diabetic autonomic neuropathy [4,5]. DBD encompasses a broad spectrum of urinary tract symptoms, ranging from hypocontractility to hypercontractility of the detrusor smooth muscle [6]. Clinical manifestations of DBD include areflexia, urinary retention, overflow incontinence, urge incontinence, and increased bladder capacity [7]. The etiology of DBD is multifactorial, involving cellular alterations and tissue damage resulting mostly from chronic hyperglycemia, oxidative stress, and microvascular damage at the level of detrusor smooth muscle and urothelium [8].

During chronic diabetes, hyperglycemia leads to an increased formation of advanced glycation end products (AGEs) [9], which are generated through the nonenzymatic reaction of dicarbonyl compounds—such as methylglyoxal (MGO), glyoxal (GO), and 3-deoxyglucosone (3-DG)—with amino acid residues, including arginine, lysine, and cysteine, in proteins, resulting in the loss of their function, and among dicarbonyl compounds, MGO is regarded as the most reactive [10]. Due to the high reactivity of these compounds, cells have developed a detoxification system located in the cytosol, the glyoxalase system, which consists of two enzymes—glutathione-dependent glyoxalase 1 (Glo1) (lactoylglutathione methylglyoxal lyase) and glyoxalase 2 (Glo2) (hydroxyacylglutathione hydrolase) [11]. These enzymes are specialized in degrading these compounds into D-lactate, which is subsequently excreted in the urine [11].

AGEs have been widely studied as key factors in a variety of diabetic complications, since glycation, the process responsible for their formation, is usually gradual and irreversible in the body, reducing the efficacy of pharmacological treatment [12]. The interaction of AGEs with their receptor (RAGE) plays a central role in several complications of diabetes, such as chronic renal failure, inflammation, atherosclerosis, cancer, and neurodegenerative diseases [13]. RAGE, a member of the immunoglobulin superfamily, has an extracellular domain with three subdomains (V, C1, and C2), a single transmembrane helix, and a cytoplasmic tail essential for intracellular activation [14]. In physiological conditions, the expression of RAGE is usually low in most tissues, but significant increases in RAGE expression can be observed in cells and tissues under the influence of hyperglycemia and accumulation of AGEs [15,16].

A previous study conducted in T2DM patients presenting lower urinary tract symptoms showed an association between increased levels of serum AGEs and bladder dysfunction [17]. In diabetic *ob*/*ob* mice, prolonged treatment with the cross-link breaker alagebrium (ALT-711) reduced the bladder levels of MGO, AGEs, and RAGE and reduced the volume per void [18]. These studies indicate that the activation of the AGE-RAGE pathway by MGO in the bladder wall contributes to the pathogenesis of diabetes-associated bladder dysfunction, but no investigation has explored RAGE as a target for therapeutic intervention for DBD. We hypothesized that RAGE blockade may constitute a novel pharmacological option to treat this disease.

TTP488 (azeliragon) is an orally bioavailable molecule that binds with high affinity to the extracellular domain of RAGE (Kd for binding to recombinant human sRAGE = 12.7 ± 7.6 nM). TTP488 has an average half-life (*t*½) of between 228 and 336 h (9.5 to 14 days) across doses in humans and has primarily been studied in neurodegenerative diseases [19]. Therefore, in the present study, we used an established model of T2DM, namely, the leptin-deficient *ob*/*ob* mouse, to evaluate the effects of the RAGE inhibitor TTP488 on bladder dysfunction. In *ob*/*ob* mice treated with TTP488, we carried out a void spot assay (in vivo) and measured the contractile responses of bladder strips (ex vivo) to verify the voiding behavior and muscle contractility, which was accompanied by measurements of levels of total AGES, fluorescent AGEs (F-AGES), MG-H1 (arginine-derived hydroimidazolone), and RAGE in serum and/or bladder tissues. Because RAGE and the glyoxalase system play critical roles in regulating dicarbonyl stress [20,21], we also investigated the protein expression and activity of Glo1, along with the activity of antioxidant enzymes, including glutathione reductase (GR), glutathione peroxidase (GPX), and superoxide dismutase (SOD), in bladder tissues.

## 2. Materials and Methods

### 2.1. Animals

Six-week-old female C57BL/6Junib (WT group) and B6.V-Lep ob/JUnib (*ob*/*ob* obese group) mice were used. The animals were obtained from CEMIB (Multidisciplinary Center for Biological Research in the Field of Laboratory Animal Science-UNICAMP, Sao Paulo, Brazil). Animal experimentation and experimental procedures were approved by the Ethics Committee in Animal Use (CEUA-UNICAMP; Protocol No. 6366-1/2023). This study followed the recommendations of the Brazilian guide for the production, maintenance or use of animals in teaching or scientific research activities/CONCEA [22] and the recommendations of the PREPARE Guidelines [23]. The animal studies reported here followed the ARRIVE guidelines [24]. Five mice were housed in polypropylene cages with 41 × 34 × 18 cm dimensions located in ventilated shelters with a constant humidity (55 ± 5%) and temperature (24 ± 2 °C) under a 12 h light–dark cycle. Animals were accommodated in these cages for two weeks before starting the treatments. Animals received standard food Nuvilab^®^ and filtered water ad libitum. A previous study by our group showed no significant differences between sexes regarding the changes observed in the bladder of obese animals [18]. Therefore, this study focused on females, owing to the greater availability of female animals.

### 2.2. Study Design with TTP488 in ob/ob Mice

The experimental design was developed using the Experimental Design Assistant (EDA) tool [25]. Ten-week-old female *ob*/*ob* mice were distributed in two groups, that is, untreated and treated with TTP488, using a randomized block experimental design with the body weight as a block. The body weight at the start of the study was 35.50 ± 4.37 g (mean ± SD) and 35 ± 2.62 (mean ± SD) for untreated and TTP488-treated, respectively. Treatment with TTP488 (azeliragon; Biorbyt, Cambridge, UK, Cat. Number orb315430) was administered exclusively to *ob*/*ob* groups, at the dose of 10 mg/kg for 15 days, as previously described [26]. TTP488 was dissolved in a solution containing 0.02% dimethylsulfoxide (DMSO) in PBS, pH 7.4, and then diluted in drinking water and administered ad libitum. Wild-type mice and untreated *ob*/*ob* mice received this vehicle only. Water intake (mL/mouse/day) was measured on the final day of the experiment.

### 2.3. Glycemic Control

Before the tests of glycemic levels, animals underwent a 3 h fasting period twice a week to minimize the effect of stress fasting. After TTP488 (or vehicle) treatment, blood samples were collected from the tail vein after a 6 h fasting period, and the insulin tolerance test (ITT) was then carried out to determine the systemic insulin sensitivity. After the first blood collection was obtained (time zero), animals received an intraperitoneal injection of regular insulin (1.00 U/Kg; Humulin R, Ely Lilly, Indianapolis, IN, USA). Blood samples were subsequently collected 5, 10, 15, 20, 25, and 30 min after the insulin injection. Glucose levels were measured using a glucometer (ACCUCHEK Performa; Roche Diagnostics, Indianapolis, IN, USA). The constant rate for blood glucose disappearance (K_Itt_) was calculated using the formula 0.693/(*t*½) × −1 × 100 [27], which is based on the linear regression of the Neperian logarithm of glucose concentrations obtained from 0 to 30 min of the test. K_Itt_ was calculated.

### 2.4. Blood Collection, Serum Processing, and Bladder Extraction

Animals were anesthetized with isoflurane at a concentration exceeding 5%, and cervical dislocation was performed to confirm euthanasia. Blood samples of 0.5 mL were collected by intracardiac puncture and centrifuged (5000× *g*) for 10 min at 4 °C, and the resulting serum was transferred to microcentrifuge tubes. The bladder was isolated and stored, frozen at −80 °C or fixed in 10% formalin for later processing.

### 2.5. Measurements of MGO-AGE-RAGE Axis

The measurement of MGO levels in both serum and bladder tissues was carried out in samples previously deproteinized using a Deproteinizing Sample Preparation Kit (TCA, Abcam, Cambridge, UK, ab204708), according to the manufacturer’s instructions. Levels of MG-H1 were measured in deproteinized samples using the fluorometric methylglyoxal assay kit (Abcam, Cambridge, UK, ab273284). Levels of total AGEs (N-ε-(carboxymethyl) lysine [CML], glyoxal-derived lysine dimer, N-ε-(1-carboxyethyl) lysine [CEL], methylglyoxal-derived lysine dimer, 3-deoxyglucosone-derived lysine dimer, and 3-deoxyglucosone) were measured in the serum and bladder using the AGE Assay Kit (Cat. No. ab238539, Abcam, Cambridge, UK) through colorimetry, following the manufacturer’s instructions. RAGE was quantified in the bladder using the Mouse RAGE ELISA Kit (Cat. No. ab100738, Abcam, Cambridge, UK), according to the manufacturer’s instructions. The F-AGEs were quantified in the serum by fluorescence using excitation and emission wavelengths of 360 nm and 447 nm, respectively, as described previously [18].

### 2.6. Measurements of Glo1 Activity and Protein Expression in Bladder Tissue

The Glo1 activity in bladder tissues was measured using the Glo1 Activity Assay Kit (Catalog No. MAK114, Sigma-Aldrich, Saint Louis, MO, USA), according to the manufacturer’s instructions. Briefly, bladders were homogenized in sodium phosphate buffer (350 μL, pH 7.0) and centrifuged (2000× *g*, 30 min, 4 °C). The cell supernatants were kept on ice until processing for the measurement of Glo1 activity. The results were normalized to the total protein in the samples.

For Glo1 protein expression, bladder tissues were initially homogenized in RIPA buffer (Catalog No. R0278, Sigma-Aldrich, Darmstadt, Germany) containing a protease inhibition cocktail (10 μL/mL; Catalog No. P8340, Sigma-Aldrich, Darmstadt, Germany), after which a total protein extract was obtained. Samples of the total protein extract were maintained at 4 °C for 1 h and then centrifuged (12,000× *g*, 15 min, 4 °C). Protein concentrations in the supernatants were determined using the DC Protein Assay Kit I (Catalog No. 5000111EDU, BioRad, Hercules, CA, USA). Samples containing 30 µg of protein were treated with 4× Laemmli buffer containing 355 mM of 2-mercaptoethanol (Catalog No. 161-0747, BioRad, Hercules, CA, USA) and then heated in a boiling water bath for 5 min and resolved by sodium dodecyl sulfate–polyacrylamide gel electrophoresis (SDS-PAGE). Using a semi-dry device (Bio-Rad, Hercules, CA, USA), the proteins were electro-transferred to a nitrocellulose membrane (20 V, 20 min). The membrane was pre-incubated overnight in blocking buffer (0.5% non-fat dried milk, 10 mM Tris, 100 mM NaCl, and 0.02% Tween 20) at 4 °C to reduce nonspecific protein binding. Primary antibodies for Glo1 (1:1000, Cat. No. ab96032, Abcam, Cambridge, UK), diluted in 3% BSA, were validated and tested according to a previous study [28]. A similar procedure was used for monoclonal β-actin peroxidase (1:50,000, Cat. No. A3854, Sigma-Aldrich, Darmstadt, Germany), instead using 1 h incubation at room temperature. Next, the membranes were incubated with the secondary antibody, HRP-linked anti-rabbit IgG (1:5000; Catalog No. 7074S, Cell Signaling Technology, Danvers, MA, USA), diluted in basal solution for 1 h. Immunoreactive bands were detected using the Clarity Western ECL Substrate (Catalog No. 1705061, BioRad, Hercules, CA, USA), an enhanced BioRad chemiluminescence system. Densitometry analysis was performed using Image Lab Software Version 6.1 (BioRad, Hercules, CA, USA). The results are represented as the ratio of protein expression relative to β-actin and to the control group (a.u.).

### 2.7. Measurement of Antioxidant Activity in the Bladder Tissue

Antioxidant activity in bladder tissues was assessed by quantifying the activity of superoxide dismutase (SOD), glutathione reductase (GR), and glutathione peroxidase (GPX) enzymes using the following commercially available kits: Superoxide Dismutase Assay Kit (Cayman Chemical, Ann Arbor, MI, USA, Cat. No. 706002), Glutathione Reductase Assay Kit (Cayman Chemical, Ann Arbor, MI, USA, Cat. No. 703202), and Glutathione Peroxidase Assay Kit (Cayman Chemical, Ann Arbor, MI, USA, Cat. No. 703102).

### 2.8. Bladder Histology for Collagen Analyses and Second Harmonic Generation (SHG) to Analyze Collagen

The bladder was fixed with 10% phosphate-buffered formalin for 24 h, dehydrated in ethanol, and embedded in paraffin. Next, 5 μm sections of bladders were made using a microtome (Leica, Wetzlar, Germany). Bladder sections were then dewaxed in xylene, rehydrated in gradient alcohol, and stained with hematoxylin and eosin (H&E) to perform collagen analysis by SHG. The SHG signal from collagen fibers was acquired using a Zeiss LSM 780-NLO confocal microscope, an upright Axio Observer Z.1 (Carl Zeiss AG, Oberkochen, Germany), equipped with a pulsed laser with a temporal duration of 100 fs and a repetition rate of 80 MHz (model Chameleon Discovery NX; Coherent Inc., Santa Clara, CA, USA). The experiment was conducted at the National Institute of Science and Technology in Applied Photonics for Cellular Biology (INFABIC) at the University of Campinas (UNICAMP, São Paulo, Brazil). The laser was directed into the microscope through a series of mirrors; passed through an acousto-optic modulator (AOM), a collimating telescope (T), and a quarter-wave plate (λ/4); and finally focused on the sample using an oil-immersion objective (EC Plan-Neofluar 40×/1.3; Carl Zeiss AG, Oberkochen, Germany). All SHG images of the samples were acquired with an excitation wavelength of 940 nm and an average power of 30 mW. The SHG signal was collected in the transmitted mode using a 0.55 NA-WD 26 mm condenser (Carl Zeiss AG, Oberkochen, Germany) and detected by the transmitted non-descanned detector (NDD,T) after passing through a filter cube containing an SP/485 filter. Meanwhile, the TPEF (Two-Photon Excitation Fluorescence) signal was acquired in the reflected mode using the reflected non-descanned detector (NDD,R), passing through a filter cube containing a mirror (Carl Zeiss, 1512-461) followed by an LP/490 filter. After excitation, the laser was blocked in both transmitted and reflected modes by an SP/690 optical filter. The acousto-optic modulator in the laser path controlled the laser power within the Zeiss system. The collimating telescope was employed to match the beam diameter to the back aperture of the objective, and the quarter-wave plate (λ/4) was used to uniformly excite the collagen fiber signal in all orientations [29,30]. All images were acquired with a field of view (FoV) of 1024 × 1024 pixels (212.55 × 212.55 µm), a pixel dwell time of 1.52 µs, and a total scan time of 15.49 s. Detector gain and offset parameters were kept constant during image acquisition to ensure consistency in quantitative analysis. The organization and intensity of collagen fibers, captured through the SHG signal, were quantified using the CurveAlign and Fiji software, respectively. CurveAlign provides a robust platform for quantifying fibrillar collagen at three levels: global, by Region of Interest (ROI), and by individual fiber. This software employs the curvelet transform to represent fiber edges and segments, offering detailed information on scale, location, and orientation. Before analyzing the organization and intensity of collagen fibers, all 24-bit images were converted to 8-bit in Fiji, standardizing the grayscale range between 0 (black) and 255 (white). SHG images were saved as TIF files and imported into CurveAlign for fiber alignment and orientation quantification. The alignment score ranges from 0 to 1, where 1 indicates perfectly aligned fibers, and 0 represents randomly distributed fibers. Relative to the horizontal axis, the orientation angle varies between 0 and 180°. For intensity analysis, brightness levels were not modified except for background subtraction (Process > Math > Subtract > Value). An automatic threshold (15–255) was then applied uniformly across all images, and a table with all preselected features was generated using the Analyze > Set Measurements menu. All metrics were saved in an Excel spreadsheet for subsequent statistical comparison between tissue samples from the experimental groups [29,30].

### 2.9. Void Spot Assay on Filter Paper

Animals, with no access to water but free access to food, were housed individually in clean cages, covered with filter paper (qualitative filter paper 250 g Unifil^®^, cod. 502.1250) measuring 25 × 15 cm [31]. The void spot assay was performed for three hours in the cage in the same period (9–10 a.m. to 12–1 p.m.) with the room temperature and humidity maintained at 24 ± 1 °C and 53 ± 1%, respectively. The filter papers were dried and imaged using UV light (Photo-documenter Chemi-Doc, Bio-Rad, Hercules, CA, USA). Filter papers were analyzed using the Fiji version of ImageJ Software (version 1.46r) (http://fiji.sc/wiki/index.php/Fiji, accessed on 26 July 2024) [32]. We analyzed (i) the urine volume in the 3 h experiment, (ii) the average volume per spot on the paper, and (iii) the number of spots on the paper, which corresponds to total urine volume (μL), volume per void (μL), and urine spot number, respectively [18].

### 2.10. Ex Vivo Functional Assays on Isolated Bladders

After the bladder removal, two longitudinal bladder strips with intact mucosa were obtained from each animal. The strips were immersed in 10 mL organ baths containing Krebs–Henseleit solution (117 mM NaCl, 4.7 mM KCl, 2.5 mM CaCl_2_, 1.2 mM MgSO_4_, 1.2 mM KH_2_PO_4_, 25 mM NaHCO_3_, and 5.5 mM glucose, pH 7.4). The Krebs–Henseleit solution was bubbled with a mixture of 95% O_2_ and 5% CO_2_ throughout the experimental procedure. Bladder strips were allowed to equilibrate for 45 min, with the bathing medium changed every 15 min. At the beginning of the experiments, the isometric force of the bladder strips was kept at 5 mN and recorded using a Power Lab system (ADInstruments Inc., Sydney, Australia). We built cumulative concentration–response curves to the muscarinic receptor agonist carbachol (1 nM–100 μM; Sigma Aldrich, MO, USA) and non-cumulative curves to the P2X1 purinergic agonist α,β-methylene ATP (1–10 μM, with 20-min intervals between concentrations to avoid tachyphylaxis; Sigma Aldrich, MO, USA). The bladder contractions in response to the depolarizing agent potassium chloride (KCl, 80 mM) were also measured on bladder strips to verify the receptor-independent responses. The contractile responses were expressed as mN per milligram of tissue (mN/mg).

### 2.11. Electrical-Field Stimulation (EFS) in Isolated Bladders

Bladder strips were placed between two platinum ring electrodes connected to a stimulator (Grass Technologies, Warwick, RI, USA). EFS at 1 to 32 Hz (80 V, 1 ms pulse width, and trains of stimuli lasting 10 s) were applied with 2 min intervals between stimulations. The contractile responses were expressed as mN/mg.

### 2.12. Statistical Analysis

The GraphPad Prism Version 6 Software (GraphPad Software, Inc., San Diego, CA, USA) was used for all statistical analyses. The parametric distribution of the data was assessed with the Shapiro test. Statistical differences between groups were determined using two-way ANOVA, followed by Bonferroni’s multiple comparisons test. All results are presented as the mean ± standard error of the mean (SEM). Results with *p*-values lower than 0.05 were considered significant.

## 3. Results

### 3.1. Effect of TTP488 Treatment on Body Weight and Glycemic Parameters

**Figure 1:**
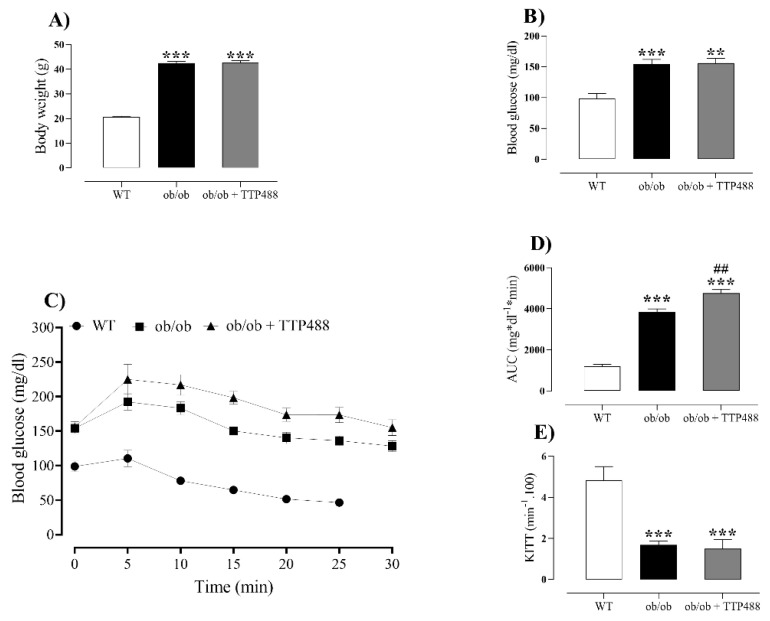
Body weight and glycemic parameters in female *ob*/*ob* mice treated with TPP488 (10 mg/kg) or receiving vehicle (0.02% DMSO in PBS) in drinking water for 15 days. Body weight (**A**), fasting blood glucose (**B**), whole-body insulin sensitivity (**C**), area under the curve (AUC) for the insulin sensitivity test (**D**), and Kitt values (**E**). Data are expressed as mean ± SEM (*n* = 17 to 20 animals for body weight in panel (**A**); and *n* = 5–7 animals per group for glycemic parameters in panels (**B**–**E**)). ** *p <* 0.01, *** *p <* 0.001 compared with the wild-type (WT) group (one-way ANOVA followed by Dunnet’s comparisons test); ## *p <* 0.01 compared with the *ob*/*ob* group (one-way ANOVA followed by Bonferroni’s multiple comparisons test).

Figure 1A,B present data on body weight and glycemic control in all groups. *ob*/*ob* mice exhibited significantly higher body weight and fasting glucose levels compared with the WT group (*p <* 0.001), both of which were unaffected by treatment with TTP488 (10 mg/kg, 15 days). Next, insulin tolerance tests were performed, when glucose levels were evaluated within 30 min after an intraperitoneal injection of insulin, allowing us to calculate both the area under the curve (AUC_0–30 min_) and Kitt values, as an index of insulin resistance. Blood glucose levels within 30 min of the insulin injection were consistently higher in *ob*/*ob* mice compared with the WT group, revealing AUC values higher in *ob*/*ob* than in the WT group (*p <* 0.001; Figure 1C,D). In *ob*/*ob* mice treated with TTP488, the AUC was significantly higher than that obtained for the untreated animals, although the difference was small (Figure 1D). The Kitt values were significantly reduced in *ob*/*ob* compared with WT mice, but no differences were found between TTP488-treated and untreated mice (Figure 1E).

### 3.2. Effect of TTP488 Treatment on the MGO-AGE-RAGE Axis in Serum and Bladder Tissues

**Figure 2:**
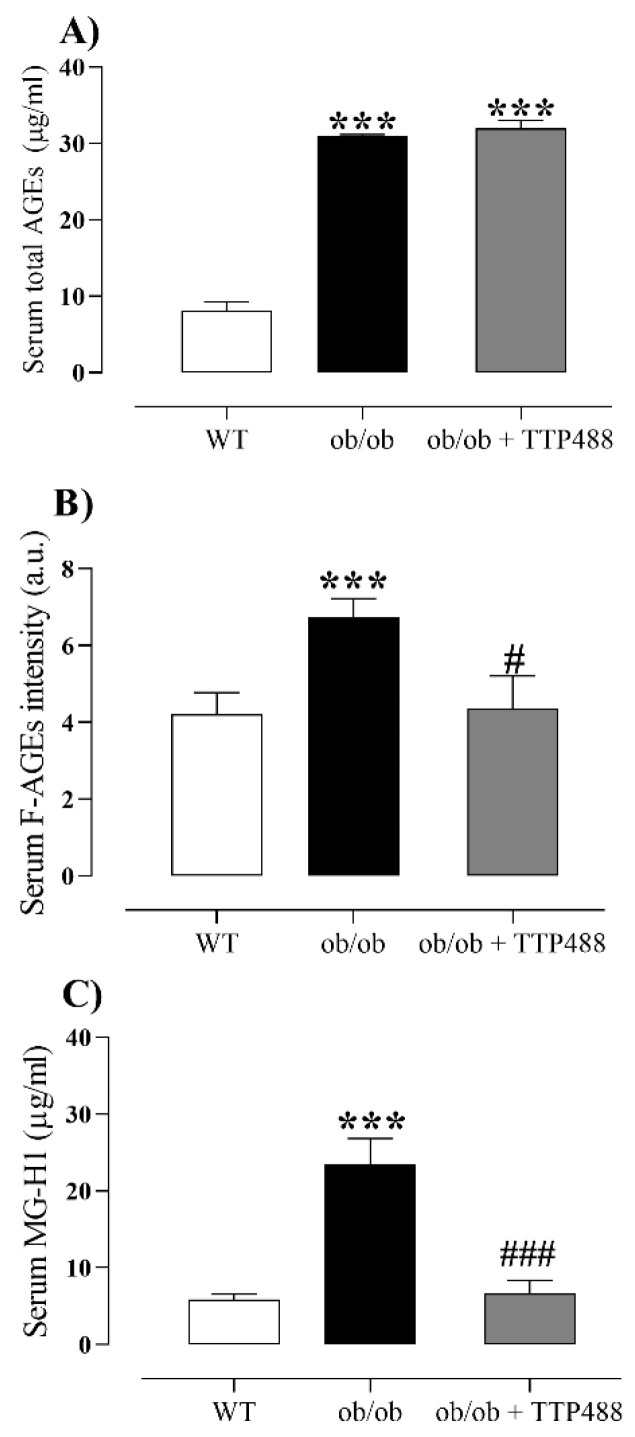
Levels of total AGEs (**A**), fluorescent AGEs (F-AGEs) (**B**), and methylglyoxal-derived hydroimidazolone (MG-H1) (**C**) in serum of female *ob*/*ob* mice treated with TPP488 (10 mg/kg) or receiving vehicle (0.02% DMSO in PBS) in drinking water for 15 days. Total AGEs in panel (**A**) consist of N-epsilon-(carboxymethyl) lysine (CML), glyoxal-derived lysine dimer (GOLD), N-epsilon-(1-carboxyethyl) lysine (CEL), methylglyoxal-derived lysine dimer (MOLD), 3-deoxyglucosone-derived lysine dimer (DOLD), and 3-deoxyglucosone (3-DG). Data are expressed as mean ± SEM (*n* = 5–7 animals for total AGEs and MG-H1 and *n* = 8–14 animals for F-AGEs). *** *p <* 0.001 compared with the wild-type (WT) group (one-way ANOVA followed by Dunnet’s comparisons test) # *p <* 0.05, ### *p* < 0.01 compared with untreated *ob*/*ob* mice (one-way ANOVA followed by Bonferroni’s multiple comparisons test).

To assess the effect of TTP488 treatment on the MGO-AGE-RAGE axis, we quantified the levels of total AGEs, fluorescent AGEs (F-AGEs), and MG-H1 in serum and bladder tissues. The levels of total AGEs (Figure 2A), F-AGEs (Figure 2B), and MG-H1 (Figure 2C) in serum were markedly higher in *ob*/*ob* mice compared with the WT group (*p <* 0.001). TTP488 treatment in *ob*/*ob* mice did not affect the levels of total AGEs (Figure 2A) but significantly reduced those of F-AGEs (*p <* 0.05; Figure 2B) and MG-H1 (*p <* 0.001; Figure 2C).

**Figure 3:**
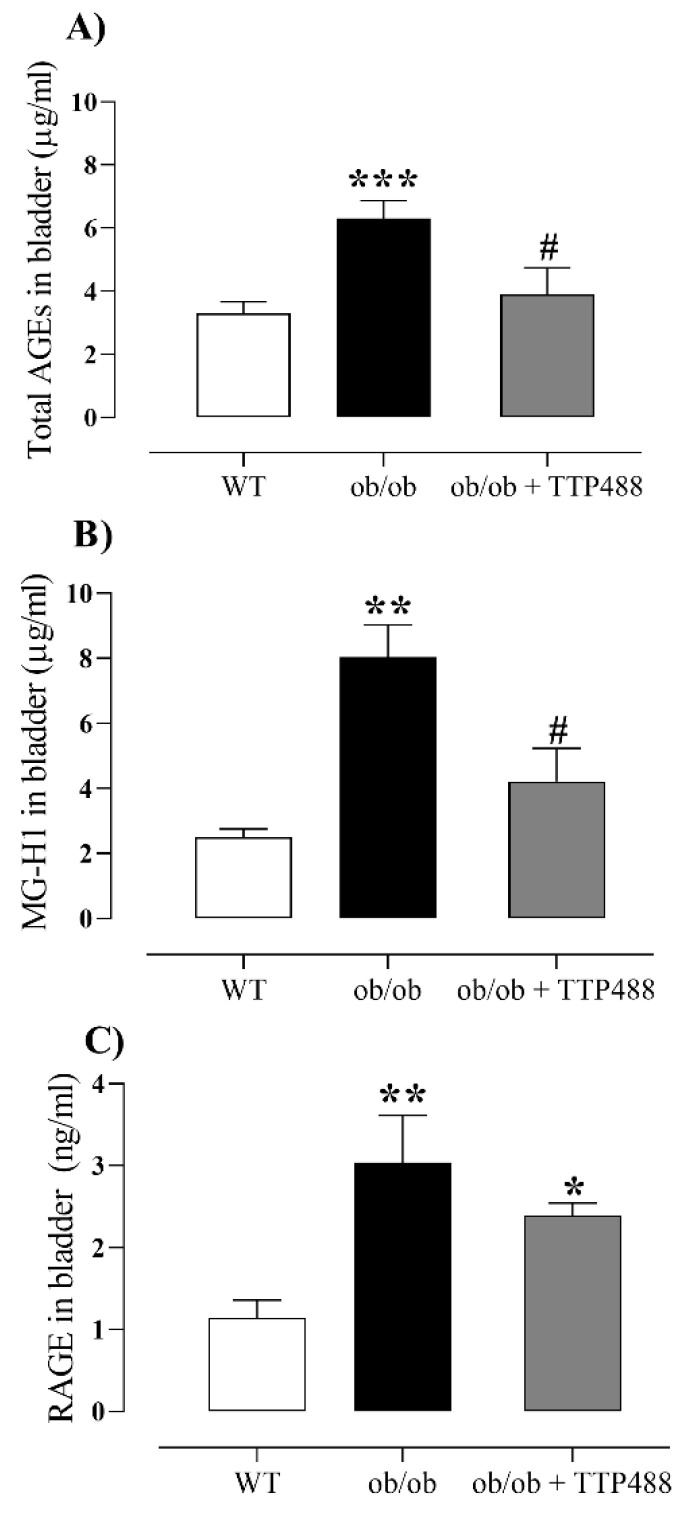
Levels of total AGEs (**A**), methylglyoxal-derived hydroimidazolone (MG-H1) (**B**), and RAGE (**C**) in bladders of female *ob*/*ob* mice treated with TPP488 (10 mg/kg) or receiving vehicle (0.02% DMSO in PBS) in drinking water for 15 days. Total AGEs in panel (**A**) consist of N-epsilon-(carboxymethyl) lysine (CML), glyoxal-derived lysine dimer (GOLD), N-epsilon-(1-carboxyethyl) lysine (CEL), methylglyoxal-derived lysine dimer (MOLD), 3-deoxyglucosone-derived lysine dimer (DOLD), and 3-deoxyglucosone (3-DG). Data are expressed as mean ± SEM (*n* = 7–15 animals in each group). * *p <* 0.05, ** *p <* 0.01, *** *p <* 0.001 compared with the wild-type (WT) group (one-way ANOVA followed by Dunnet’s comparisons test); # *p <* 0.05 compared with the untreated *ob*/*ob* group (one-way ANOVA followed by Bonferroni’s multiple comparisons test).

Similarly to serum, the levels of total AGEs and MG-H1 in bladder tissues were significantly higher in *ob*/*ob* mice compared to the WT group (*p <* 0.01) and were restored to the levels of the WT group by TTP488 treatment (Figure 3A,B). We next evaluated RAGE protein levels in the bladder tissues (Figure 3C). RAGE levels were significantly higher in *ob*/*ob* mice compared with the WT group (*p <* 0.01), but TTP488 treatment failed to significantly affect this protein level in this group (Figure 3C). When the levels of total AGEs and MG-H1 in serum were compared to those in bladder tissues, we observed that the levels of both markers in serum were 4.8-fold and 2.6-fold higher than those in bladder tissues (*p <* 0.001).

### 3.3. Effect of TTP488 Treatment on Glo1 Protein Expression and Activity and on Antioxidant System in Bladder

**Figure 4:**
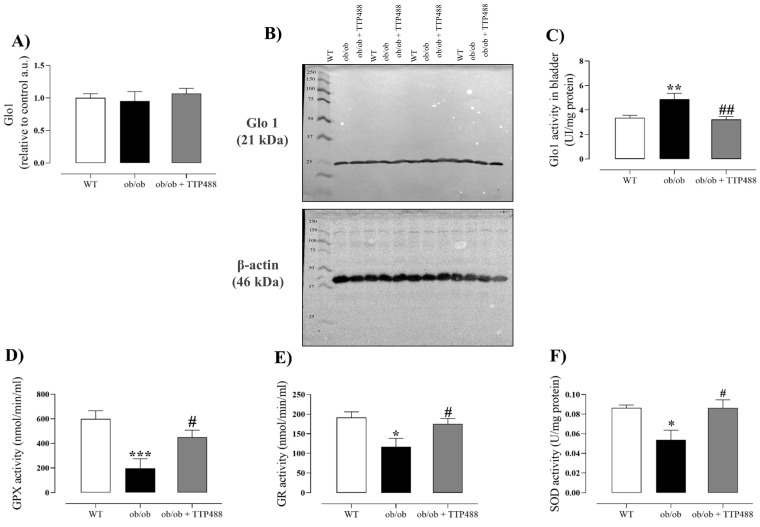
Analysis of protein levels (**A**,**B**) and activity of glyoxalase 1 (Glo1) (**C**) and enzyme activities of glutathione reductase (GR) (**D**), glutathione peroxidase (GPX) (**E**), and superoxide dismutase (SOD) (**F**) in the bladders of female *ob*/*ob* mice treated with TPP488 (10 mg/kg) or receiving vehicle (0.02% DMSO in PBS) in drinking water for 15 days. Data are expressed as mean ± SEM (*n* = 5 animals in each group for Western blotting analysis, and *n* = 6–7 animals for enzymatic activity analysis). * *p* < 0.05, ** *p* < 0.01, *** *p* < 0.001 compared with the wild-type (WT) group (one-way ANOVA followed by Dunnet’s comparisons test); # *p* < 0.05, ## *p* < 0.01 compared with the untreated *ob*/*ob* group (one-way ANOVA followed by Bonferroni’s multiple comparisons test).

Glo1 protein expression and activity were evaluated in the bladders of animals treated or not with TTP488. No significant differences in Glo1 protein expression were observed among the WT, *ob*/*ob*, and *ob*/*ob* + TTP488 groups (Figure 4A,B). However, Glo1 enzymatic activity was significantly higher in the bladder tissues of *ob*/*ob* mice compared with the WT group (*p <* 0.01) and was normalized by TTP488 treatment, bringing the enzyme activity to the levels of the WT group (Figure 4C).

Antioxidant activity in the bladder was assessed by quantifying the enzyme activities of GR (Figure 4D), GPX (Figure 4E), and SOD (Figure 4F). Our data showed significant reductions in all these enzymes’ activities in *ob*/*ob* mice compared with the WT group (*p <* 0.05), and they were all restored by TTP488 treatment, achieving the same activity levels as the WT group (Figure 4D–F).

### 3.4. Effect of TTP488 Treatment on Collagen

**Figure 5:**
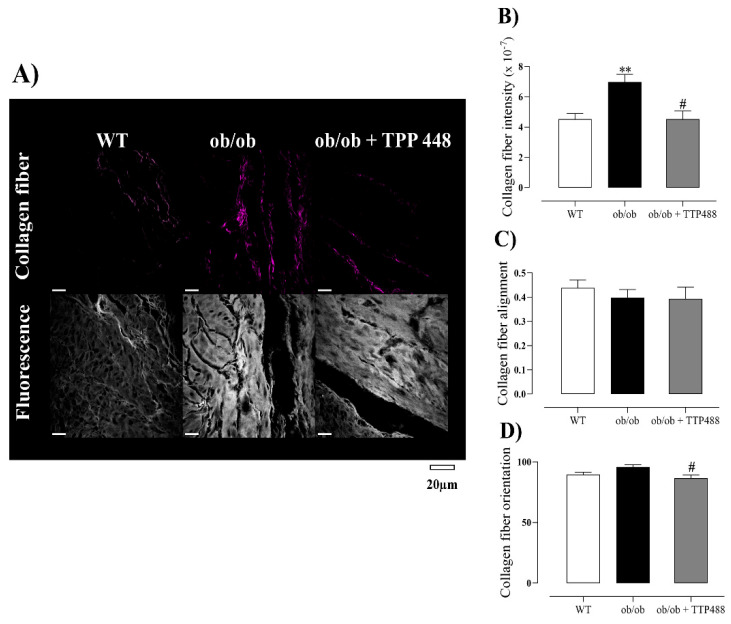
Analysis of the organization and intensity of collagen fibers in the detrusor of female *ob*/*ob* mice receiving either vehicle (0.02% DMSO in PBS) or TPP488 (10 mg/kg) in drinking water for 15 days in comparison with untreated female wild-type (WT) mice. Images were obtained from H&E-stained slides, whereas collagen analysis was performed using the second harmonic generation (SHG), and representative images are shown in panel (**A**). Parameters of collagen analyses, intensity (**B**), alignment (**C**), and organization (**D**). Data are expressed as mean ± SEM (*n* = 5 each group). ** *p <* 0.01 compared with the WT group; # *p <* 0.05 compared with the untreated *ob*/*ob* group (one-way ANOVA followed by Bonferroni’s multiple comparisons test).

To identify potential structural changes in collagen fiber resulting from obesity and diabetes, we quantified collagen in the bladder tissues using the SHG technique, which allows for the analysis of fiber intensity, alignment, and orientation. Representative images are shown in Figure 5A. Our data revealed a significant increase in collagen intensity in the *ob*/*ob* group compared with the WT group (*p* < 0.01), an effect significantly reversed by TTP488 treatment (*p* < 0.05; Figure 5B). The fiber alignment showed no differences among the three groups studied (Figure 5C), whereas the fiber orientation was slightly reduced (although significantly at *p* < 0.05) in *ob*/*ob* mice treated with TTP488 compared with the untreated *ob*/*ob* group (Figure 5D).

### 3.5. Effect of TTP488 Treatment on Voiding Behavior Assessed by Filter Paper Assay and Water Consumption

**Figure 6:**
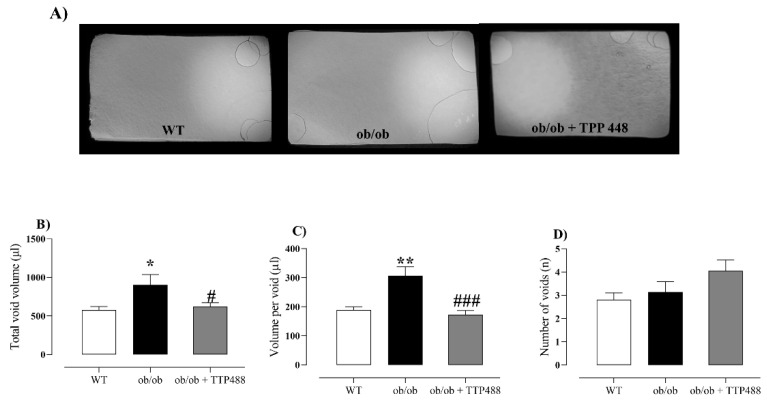
Void spot analysis on filter paper in female wild-type (WT) mice, *ob*/*ob* mice receiving vehicle (0.02% DMSO in PBS, 15 days), and *ob*/*ob* mice treated with TPP488 (10 mg/kg, 15 days). Panel (**A**) shows representative images of the void spot assays. Panels (**B**–**D**) show the total void volume, volume per void, and number of voids, respectively. Data are expressed as mean ± SEM (*n* = 14–18 animals per group). * *p <* 0.05, ** *p <* 0.01 compared with the WT group; # *p <* 0.05, ### *p <* 0.001 compared with untreated *ob*/*ob* mice (one-way ANOVA followed by Bonferroni’s multiple comparisons test).

Representative images of the voiding records in all groups studied are shown in Figure 6A. Compared with the WT group, *ob*/*ob* mice showed significant increases in both total void volume (*p <* 0.05; Figure 6B) and volume per void (*p <* 0.01; Figure 6C), with no significant alterations in the number of voids (Figure 6D). TTP488 treatment abrogated the increases in total void volume (*p <* 0.05; Figure 6B) and volume per void (*p <* 0.001; Figure 6C), without affecting the number of voids (Figure 6D). Water consumption was measured at the end of the experiment (mL/mice/day), showing rates of 6.06 ± 1.14, 6.30 ± 1.55, and 6.03 ± 1.33 mL/mice/day for the WT, *ob*/*ob*, and *ob*/*ob* + TTP488 groups, respectively, with no statistical differences between them (*p =* 0.99).

### 3.6. Effect of TTP488 Treatment on Ex Vivo Detrusor Contractility

**Figure 7:**
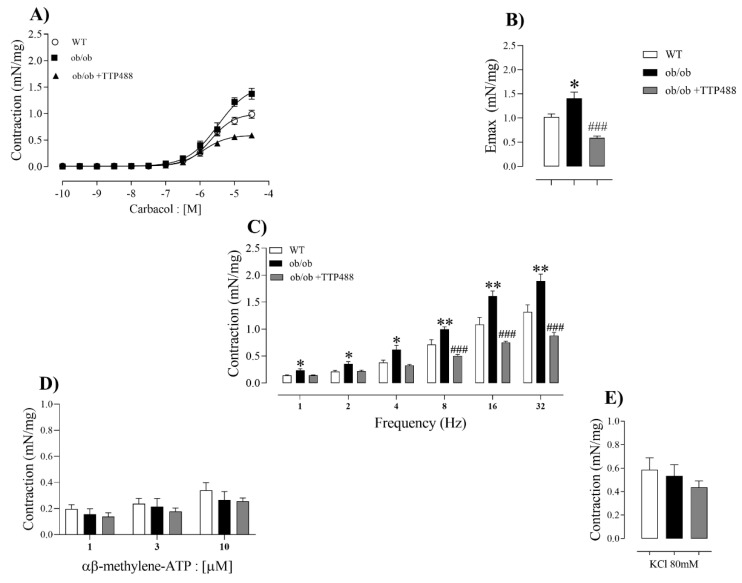
Ex vivo bladder contractions in response to carbachol (0.1 nM to 30 µM) (**A**,**B**), electrical-field stimulation (EFS; 1 to 32 Hz) (**C**), α, β-methylene-ATP (1, 3, and 10 µM) (**D**), and potassium chloride (KCl, 80 mM) (**E**). Bladder strips were obtained from female wild-type (WT) mice, ob/ob mice receiving vehicle (0.02% DMSO in PBS, 15 days), and ob/ob mice treated with TPP488 (10 mg/kg, 15 days). Data are expressed as mean ± SEM (*n* = 5–8 each group). * *p <* 0.05, ** *p <* 0.01 compared with the WT group; ### *p <* 0.001 compared with untreated ob/ob mice (one-way ANOVA followed by Bonferroni’s multiple comparisons test).

Detrusor contractility was assessed in response to the muscarinic agonist carbachol (0.1 nM to 30 µM; Figure 7A,B), electrical-field stimulation (EFS, 1 to 32 Hz; Figure 7C), the purinergic P2X1 agonist α, β-methylene ATP (1 to 10 µM; Figure 7D), and a depolarizing KCl solution (80 mM; Figure 7E).

The *ob*/*ob* group exhibited a significant increase in contractile responses to carbachol and EFS (*p* < 0.05), but not to α, β-methylene ATP or KCl. Treatment of *ob*/*ob* mice with TTP488 markedly reduced carbachol-induced detrusor contractions compared to the *ob*/*ob* group (*p* < 0.05) but not to α, β-methylene ATP or KCl. Treatment of *ob*/*ob* mice with TTP488 markedly reduced carbachol-induced detrusor contractions compared to the *ob*/*ob* group (*p* < 0.05), while no significant difference was detected between the *ob*/*ob* + TTP488 and WT groups (*p =* 0.07). With TTP488 treatment, a significant reduction in the response to EFS was observed at frequencies from 8 Hz onwards (*p* < 0.001) compared to the *ob*/*ob* group, with no difference relative to the WT group. The contractile responses to α, β-methylene ATP and KCl remained unaffected by TTP488 treatment.

## 4. Discussion

In this study, we employed an established model of T2DM and obesity (*ob*/*ob* mouse) to explore the involvement of the MGO-AGE-RAGE axis in DBD, focusing on the ability of the RAGE inhibitor TTP488 (azeliragon) to reduce the functional and molecular alterations observed in the bladder tissues. We found that two-week oral treatment with TTP488 (10 mg/kg) significantly reduced both the total void volume and volume per void in *ob*/*ob* mice (void spot assays), as well as the hypercontractile detrusor muscle response to carbachol and EFS. TTP488 treatment did not significantly affect RAGE expression but normalized the high MG-H1 levels (the main MGO-derived AGE) and Glo-1 activity and reduced the enzyme activities of GR, GPX, and SOD in the bladder tissues of *ob*/*ob* mice to the levels of WT animals.

At 12 weeks old, female *ob*/*ob* mice displayed significant weight gain and persistent hyperglycemia, together with marked insulin resistance (Kitt index), thus confirming the diabetic state of the animals. Water consumption was not modified in *ob*/*ob* mice compared with the WT group, which is consistent with previous studies showing that this parameter increases up to 4 weeks old, declining thereafter [33]. Additionally, hyperglycemia/obesity was strongly associated with significant activation of the MGO-AGE-RAGE pathway in bladder tissues, as indicated by the elevated levels of total AGEs, MG-H1, and Glo1 activity, along with a decreased antioxidant system, as evidenced here by the reduced enzyme activities of GR, GPX, and SOD. These findings reinforce the *ob*/*ob* mouse as an appropriate model for investigating the MGO-AGE-RAGE pathway in diabetes-related bladder dysfunction [18,34].

Bladder dysfunction in *ob*/*ob* mice is characterized by increased urine output, as evidenced in the void spot assay on filter paper [18,35]. Here, we confirmed the higher total void volume and volume per void in *ob*/*ob* mice, with no significant alterations in the number of voids. In vitro bladder contractions in response to EFS result in frequency-dependent contractions in rodents, mostly due to acetylcholine and ATP release from parasympathetic nerve fibers that act on post-synaptic muscarinic M3 and P2X1 membrane receptors to produce contractions, respectively [36,37]. We found a higher contractile response to both EFS and carbachol in the *ob*/*ob* group compared to the WT group, whereas the responses to α,β-methylene ATP remained unchanged between the *ob*/*ob* and WT groups, strongly suggesting that acetylcholine may be the principal excitatory transmitter affected in this model. In addition, our findings that bladder contractions induced by the depolarizing agent KCl (80 mM) did not differ between ob/ob and WT mice indicate that the muscle force remains intact in the bladder smooth muscle of the diabetic animals, reinforcing that hypercon-tractility in response to EFS and carbachol are possibly attributed to alterations at the level of autonomic parasympathetic innervation and/or post-junctional muscarinic receptors, as previously reported in other diabetes models [38,39,40,41].

In the present work, we hypothesized that RAGE may be directly implicated in the DBD of *ob*/*ob* mice. Although RAGE binds to multiple ligands and activates various intracellular signaling pathways, it is particularly important in pathological conditions where an excess of AGEs is present, such as T2DM [42,43]. Moreover, RAGE and the glyoxalase system play critical roles in regulating dicarbonyl stress [21]. RAGE serves as a central mediator by binding to AGEs, hence initiating different intracellular processes, including oxidative stress [44,45], whereas the glyoxalase system is essential for detoxifying AGE precursor compounds, such as MGO [46], with Glo-1 regarded as a rate-limiting factor for the efficiency of this system [47].

However, the literature shows that the interaction between AGEs-RAGE and the Glo-1 system can largely vary depending on the model employed. For instance, the kidneys of obese mice on a high-fat diet with elevated levels of AGEs exhibited increased RAGE protein expression without changing Glo-1 levels [48], whereas in aortic rings from streptozotocin-induced diabetic male Sprague-Dawley rats, RAGE levels were increased while Glo-1 expression and activity were decreased [49]. In the hippocampus of streptozotocin-induced diabetic Wistar rats, both RAGE expression and Glo-1 activity were increased [50].

In our study, higher Glo-1 activity in the bladders of *ob*/*ob* was observed. Although the higher Glo-1 activity in the bladders of *ob*/*ob* mice indicates a higher efficiency of this system in detoxifying the excess of MGO, we found that the levels of MG-H1 remained elevated in serum and bladder tissues, pointing to a dicarbonyl imbalance where AGE production largely exceeds the detoxification capacity of Glo-1. Indeed, *ob*/*ob* mice are hyperphagic, explaining, at least in part, their obesity [51]. The increased levels of AGEs in serum and tissues in these animals may therefore reflect endogenous production as a result of hyperglycemia, together with the high intake of AGEs present in the animal diet.

TTP488, also referred to as azeliragon, is an orally bioavailable drug presenting high affinity to the extracellular domain of RAGE [19], but there exist few studies investigating its use in disease models [51,52]. The present study is the first to investigate the use of TTP488 in diabetes-associated bladder dysfunction. Our primary objective was to determine whether TTP488 could mitigate in vivo voiding dysfunction and ex vivo bladder hypercontractility in *ob*/*ob* mice. Our findings revealed that two-week treatment with TTP488 in *ob*/*ob* mice nearly restored the total void volume and volume per void to control levels. In addition, TTP488 abrogated the ex vivo bladder hypercontractility in response to carbachol and EFS, all suggesting that this RAGE inhibitor could be thought of as a potential therapeutic agent in the management of diabetes-associated bladder dysfunction. We then examined the effects of TTP488 treatment on the levels of total AGEs, MG-H1, and RAGE in the serum and/or bladders of obese animals. Our data revealed that TTP488 nearly suppressed the high levels of both total AGEs and MG-H1 in the bladder tissues of *ob*/*ob* mice, as well as those of F-AGEs in serum. The inability of TTP488 to reduce the levels of total AGEs in serum may be related to the high levels reached in this compartment (~30 µg/mL), being approximately 5-fold higher than those in the tissue compartment (~6 µg/mL). Moreover, the high levels of RAGE in the bladder tissues of *ob*/*ob* mice were not significantly affected by TTP488 treatment, which is not surprising, provided that this compound binds to the RAGE receptor without theoretically influencing the production of AGEs, but then what explains the reduced levels of total AGEs and MG-H1 with TTP488 treatment? We still do not have any clue on this subject but wonder whether TTP488 also acts by scavenging AGEs or breaking preformed AGEs in a similar fashion to the thiazolium compound alagebrium chloride, which can break the nonenzymatic bonds between AGEs and proteins, being also capable of directly scavenging MGO [53,54,55,56].

As the activation of the AGE-RAGE axis involves oxidative stress, the ability to neutralize reactive oxygen species (ROS) may serve as a primary defense mechanism [57]. Therefore, we investigated the antioxidant activity of the hydrogen peroxide (H_2_O_2_)-detoxifying enzyme, SOD, as well as the enzymes involved in glutathione (GSH) recycling, GR and GPX. We explored whether treatment with TTP488 could modulate the activity of these enzymes, thereby contributing to a reduction in ROS production and, consequently, a lower activation of the AGE-RAGE axis. GR plays a critical role in recycling reduced GSH, enabling its reuse to control intracellular oxidative stress [58]. When GSH scavenges ROS, it is oxidized to form oxidized glutathione (GSSG), which is subsequently converted back to its reduced, active form through the action of GR [58]. SOD catalyzes the conversion of the superoxide anion (O_2_^−^) into H_2_O_2_ and molecular oxygen [59], while GPX reduces H_2_O_2_ and lipid hydroperoxides, using GSH as a cofactor to convert H_2_O_2_ into water and oxygen [60]. Together, these enzymes play a pivotal role in regulating the cellular redox state. Reduced levels of GSH, concomitant with elevated levels of AGEs and ROS, have been reported in the blood, plasma, red blood cells, and monocytes of T2DM patients [61,62]. Therefore, we evaluated bladder antioxidant activity by analyzing the enzyme activity of GR, GPX, and SOD in the bladders of *ob*/*ob* animals. We observed that GR, GPX, and SOD activities were significantly reduced in the bladders of *ob*/*ob* mice, indicative of a prooxidative state. TTP488 treatment reduced MG-H1 levels, with the concomitant restoration of Glo-1, GR, GPX, and SOD activity in the bladders of *ob*/*ob* mice. However, an apparent paradox arises from the increased Glo-1 activity observed in the bladders of *ob*/*ob* mice alongside decreased GR activity. This can be explained by the fact that Glo-1 activity depends on the availability of GSH, while GR is responsible for converting GSSG back into GSH, potentially leading to a lower release of GSH. In the glyoxalase system, Glo-1 converts dicarbonyl compounds into less reactive intermediates with the participation of Glo-2 [63,64]. In this system, GSH regeneration is mediated by Glo-2, which hydrolyzes S-D-lactoylglutathione—produced by Glo-1 from hemithioacetal—into D-lactate, preserving its GSH cofactor for Glo-1 activity [21]. Glo-1 and Glo-2 activity was found to be increased in the bladder tissues of *ob*/*ob* mice [18]. Nevertheless, the imbalance between Glo-1 and antioxidant enzymes in the bladders of *ob*/*ob* mice was mitigated by TTP488 treatment, which restored the redox balance and reduced the levels of MG-H1 and AGEs in this tissue.

Finally, we investigated the effects of TTP488 treatment on collagen organization and intensity in the bladder using SHG microscopy, as RAGE has been implicated in the regulation of the expression of collagen type I and III genes and proteins in fibroblasts [65]. Additionally, cardiac fibroblasts derived from diabetic *db*/*db* mice exhibit greater migratory capacity compared to those from non-diabetic animals [66]. Previously, we demonstrated that prolonged exposure to MGO increases RAGE expression and total collagen in the detrusor smooth muscle [67], and that treatment with the AGE breaker alagebrium significantly reduced collagen expression in this tissue in *ob*/*ob* mice [18]. The SHG technique is a non-invasive and highly specific method for fibrillar collagen, enabling the assessment of its structural organization and abundance in tissue [68]. SHG signal intensity correlates with biological characteristics of collagen, such as fiber diameter, total content, cross-linking, degradation, and matrix organization [69,70,71,72], whereas changes in fiber orientation reflect early degenerative processes [72]. In this study, the bladders of *ob*/*ob* mice exhibited damage and degeneration of collagen fibers, as evidenced by the increased collagen signal intensity, which is consistent with a previous study showing that AGE accumulation causes RAGE-dependent collagen disruption in the intervertebral discs of mice fed a diet rich in AGEs [72]. TTP488 treatment significantly reduced both collagen signal intensity and fiber orientation, which may be linked to reduced levels of total AGEs and MG-H1 and, consequently, the inhibition of RAGE signaling in the bladder.

This study provides new insights into the role of RAGE in bladder dysfunction within the T2DM model, reinforcing its significance in diabetic complications. Building on these findings, future research should explore RAGE signaling in a DM type 1 (T1DM) model to determine whether RAGE expression is similarly increased in the bladder or whether notable differences exist when compared to T2DM. Furthermore, understanding whether RAGE antagonists could be effective in treating DBD in a T1DM model may pave the way for innovative therapeutic strategies. These investigations would contribute to a broader understanding of the mechanisms underlying bladder dysfunction in diabetes, potentially guiding the development of targeted interventions.

## 5. Conclusions

This study is the first to reveal that the selective RAGE inhibitor TTP488 improves the functional and molecular bladder alterations in obese diabetic *ob*/*ob* mice through normalizing the dysfunctional MGO-AGE-RAGE axis in bladder tissue. We propose that RAGE blockade could serve as a promising therapeutic strategy for managing diabetic bladder dysfunction.

## Data Availability

The metadata and data from this study will be available in the Unicamp Research Data Repository. Oliveira AL de, Medeiros ML de, Antunes E. 2025 (FAPESP EAA-SÃOPRF). Data related to the project “Role of the receptor for Advanced Glycation End Products (AGEr) in diabetic bladder dysfunction: A new therapeutic target?” [Internet]. DRAFT VERS. Repositório de Dados de Pesquisa da Unicamp; available from https://doi.org/10.25824/redu/VYNGJ7.

## Abbreviations

The following abbreviations are used in this manuscript:

3-DG	3-Deoxyglucosone
AGEs	Advanced glycation end products
CEL	N-epsilon-(1-carboxyethyl) lysine
CML	N-epsilon-(carboxymethyl) lysine
DBD	Diabetic bladder dysfunction
DM	Diabetes Mellitus
T1DM	Diabetes Mellitus phenotypes, with type 1
T2DM	Diabetes Mellitus phenotypes, with type 2
DOLD	3-deoxyglucosone-derived lysine dimer
EFS	Electrical-field stimulation
F-AGES	Fluorescent AGEs
Glo1	Glyoxalase 1
Glo2	Glyoxalase 2
GOLD	Glyoxal-derived lysine dimer
GO	Glyoxal
GPX	Glutathione peroxidase
GR	Glutathione reductase
GSH	Glutathione
GSSH	Oxidized glutathione
KCL	Potassium chloride
MG-H1	Methylglyoxal-derived hydroimidazolone
MGO	Methylglyoxal
MOLD	Methylglyoxal-derived lysine dimer
RAGE	Receptor for advanced glycation end products
SOD	Superoxide dismutase
SHG	Second harmonic generation
